# Polymer Materials for Optoelectronics and Energy Applications

**DOI:** 10.3390/ma17153698

**Published:** 2024-07-26

**Authors:** Ju Won Lim

**Affiliations:** George W. Woodruff School of Mechanical Engineering, Georgia Institute of Technology, 495 Tech Way, NW, Atlanta, GA 30318, USA; jlim339@gatech.edu

**Keywords:** organic materials, nanomaterials, material properties, physical analysis, optoelectronic devices, photonic devices, photovoltaics, organic light-emitting diodes (OLEDs), phototransistors

## Abstract

This review comprehensively addresses the developments and applications of polymer materials in optoelectronics. Especially, this review introduces how the materials absorb, emit, and transfer charges, including the exciton–vibrational coupling, nonradiative and radiative processes, Förster Resonance Energy Transfer (FRET), and energy dynamics. Furthermore, it outlines charge trapping and recombination in the materials and draws the corresponding practical implications. The following section focuses on the practical application of organic materials in optoelectronics devices and highlights the detailed structure, operational principle, and performance metrics of organic photovoltaic cells (OPVs), organic light-emitting diodes (OLEDs), organic photodetectors, and organic transistors in detail. Finally, this study underscores the transformative impact of organic materials on the evolution of optoelectronics, providing a comprehensive understanding of their properties, mechanisms, and diverse applications that contribute to advancing innovative technologies in the field.

## 1. Introduction

Polymeric materials have grown unprecedentedly over the last 20 years, primarily due to breakthroughs in material design synthesis and purification techniques [1,2,3]. Particularly since the mid-1900s, when groundbreaking discoveries like the emergence of conducting materials and electroluminescence in molecular crystals were made, this foundational research has been instrumental in driving significant technological advancements. These discoveries shifted attention to organic materials, demonstrating their enormous potential for electronic applications and paving the way for further technological developments [4,5]. Furthermore, an environment of collaborative research has been fostered by the interdisciplinary nature of organic optoelectronics, which encompasses disciplines such as chemistry, physics, materials science, and engineering. In addition to encouraging creativity, this blending of diverse disciplines has enabled the development of innovative materials and devices with unprecedented capabilities. As an example, the incorporation of organic materials into electronics has produced lightweight, flexible, and eco-friendly substitutes for traditional silicon-based electronics. This interdisciplinary approach has been instrumental in advancing the field and expanding the horizon of potential applications, particularly through the tunability of organic materials via synthesis.

**Figure 1:**
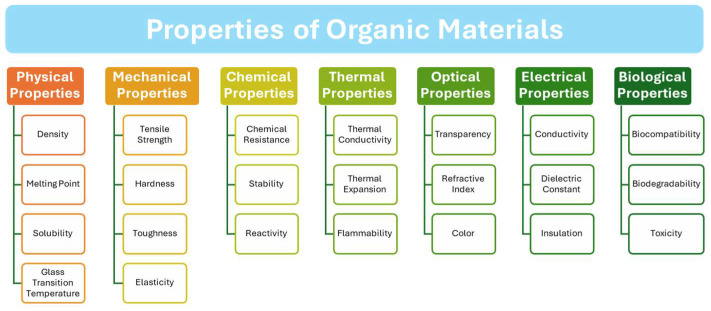
Diagram illustrating the various characteristics of polymers [18].

The tunability of organic materials through chemical synthesis offers substantial benefits [6,7]. Researchers can design and synthesize new materials with tailored properties to meet specific application requirements. This capability to engineer the molecular structure of organic semiconductors allows precise control over various properties of organic materials, such as the physical, mechanical, chemical, thermal, optical, electrical, and biological properties (Figure 1), thereby enabling the development of highly specialized devices. The customization potential of organic materials is a driving force behind their increasing popularity and widespread adoption across various applications. This progress has further fueled innovations in optoelectronics and energy applications, characterized by continuous advancements in understanding optical and electronic properties. Furthermore, a particularly promising aspect of organic optoelectronics lies in its potential for large-scale, cost-effective production. Solution-processable organic semiconductors can be deposited using versatile techniques such as spin-coating, inkjet printing, and roll-to-roll processing [8,9]. These methods enable the fabrication of electronic devices over large areas and on flexible substrates, opening new avenues for applications in wearable electronics, flexible displays, and smart packaging [10,11,12]. The ability to manufacture devices at scale and relative affordability confers a significant advantage, making organic optoelectronics highly attractive for various commercial applications.

The field of organic optoelectronics continues to evolve with ongoing innovation in material design, synthesis methodologies, and processing techniques [13,14]. Recent advances have propelled organic optoelectronics into a new era of technological prowess. As research in this domain expands, the potential applications of organic materials are becoming increasingly diverse and impactful. Presently, organic materials find extensive use in applications such as organic thin-film transistors (OTFTs), organic light-emitting diodes (OLEDs), solar cells, sensors, and photorefractive (PR) devices [15,16,17]. Their versatility, combined with their suitability for low-cost, large-area, and flexible substrate applications, positions them favorably for a broad range of technological innovations. The significant interest and robust body of research in this field underscore its importance and the exciting opportunities it presents for advancing technology and benefiting society.

This review aims to provide a comprehensive background of organic materials and an overview of the current status and future potential of semiconducting materials and organic-based optoelectronic applications, which offer distinct advantages and have played a pivotal role in the field’s development. These materials offer unique benefits for large-area device fabrication due to their flexibility and compatibility with various processing techniques, enabling the creation of high-performance single-component optoelectronic devices. Noteworthy examples include bulk heterojunction organic photovoltaics, organic light-emitting diodes (OLEDs), photorefractive (PR) devices, and polymer-based phototransistors. Recent advances in polymer design, synthesis, and processing have led to significant enhancements in the performance of polymer-based devices, underscoring the vast potential of these materials in driving future technological advancements.

## 2. Advancements and Applications of Polymer Organic Materials

### 2.1. Organic Materials

Polymer organic materials represent a dynamic and promising category within materials science, distinguished by their carbon-based molecular structures and versatile properties essential for a wide array of applications. These materials are characterized by their tunable electronic and optical properties, which can be precisely adjusted through molecular design and chemical synthesis, allowing researchers to control critical parameters such as energy levels, bandgaps, and charge carrier mobility for specific technological needs [19]. This capability not only supports cost-effective manufacturing but also facilitates their integration into flexible and stretchable electronic devices, where mechanical adaptability is crucial.

**Figure 2:**
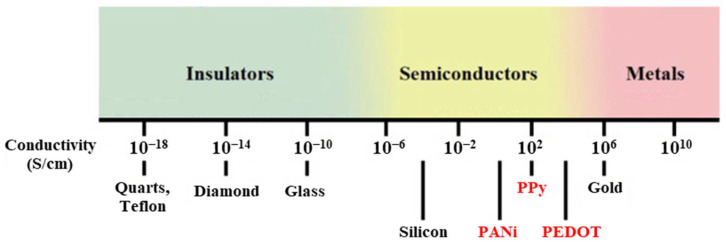
Conductivity of conjugated polymer materials in comparison to other typical materials. The commonly used conducting polymers are highlighted in red. This figure is adapted from [29] (reproduced under terms of the CC-BY license).

The conductivity of polymer materials varies widely, spanning from insulators to semiconductors and even approaching that of metals in some cases. Traditional polymer materials are typically insulators with very low electrical conductivity due to their lack of free charge carriers and extended π-conjugation. For example, polyethylene and polystyrene exhibit conductivities in the range of 10^−16^ to 10^−18^ S/cm, which is characteristic of insulating materials [20,21,22]. In contrast, semiconducting polymers such as polyaniline (PANi), polypyrrole (PPy), and poly(3,4-ethylenedioxythiophene) (PEDOT) exhibit a wide range of conductivities depending on doping, processing methods, and specific formulations (Figure 2). For instance, the conductivity of PANi typically ranges from 1 to 10 S/cm, varying with processing conditions [23], while the conductivity of PPy spans from 1 to 10^2^ S/cm [24,25]. The conductivity of PEDOT, which is among the most commonly used conducting polymers, can reach approximately 10^2^ to 10^3^ S/cm [26,27]. This semiconducting behavior is attributed to their conjugated π-electron systems, which facilitate charge transport. When heavily doped, some conductive polymers, like polyaniline and polypyrrole, can achieve conductivities on the order 10^3^ S/cm or higher, approaching those of metallic conductors such as copper or aluminum, which exhibit conductivities around 10^6^ S/cm [28,29]. This remarkable tunability in conductivity, combined with the inherent mechanical flexibility and processability of polymer materials, enables their use in a diverse range of applications from flexible electronics to advanced sensing technologies.

Temperature significantly influences the properties and performance of organic polymers. As the temperature increases, the mobility of polymer chains generally rises, leading to enhanced ductility and flexibility but potentially reduced strength. At elevated temperatures, polymers may undergo phase transitions, such as glass transition or melting, which can affect their mechanical and thermal stability. Conversely, low temperatures can cause polymers to become brittle and more susceptible to cracking. Additionally, temperature affects the diffusion rates of small molecules within the polymer matrix, impacting processes such as permeation and degradation. Understanding these temperature effects is crucial for optimizing the application and durability of organic polymers in various environments.

Mechanically, organic polymers exhibit a broad range of properties depending on their molecular structure and processing conditions, ranging from soft and elastomeric to rigid and thermally stable [30]. For example, polyethylene is widely used for its flexibility and toughness in packaging, while polyimides are chosen for their high thermal stability and rigidity in aerospace applications [31,32]. This versatility makes them suitable for diverse applications in structural components, packaging materials, and biomedical devices. Biocompatibility is another significant attribute of polymer organic materials, essential for biomedical applications such as drug-delivery systems, tissue engineering scaffolds, and implantable medical devices [33,34]. Some polymers can be engineered to promote specific interactions with biological systems, thereby enhancing their effectiveness and safety in medical applications. Material properties, such as physical properties, mechanical properties, chemical properties, and optical properties of representative polymers, are provided in Table 1.

Efforts to enhance the environmental stability and durability of organic polymers are critical in research and development. Strategies include surface modifications, encapsulation techniques, and polymer blending to improve resistance to factors such as moisture, oxygen, UV radiation, and mechanical wear [41]. These efforts are crucial for extending the operational lifetime of devices based on organic materials and ensuring their reliability in real-world conditions. Certain conjugated polymers within this class also exhibit semiconducting behavior, making them suitable for applications in electronic devices such as organic field-effect transistors (OFETs), sensors, and actuators. The ability to control charge transport properties through chemical doping or structural modifications opens avenues for developing high-performance electronic components using organic materials. Optically, polymer organic materials can be engineered to possess transparency across a broad spectrum, from ultraviolet to near-infrared wavelengths, which is valuable for applications requiring optical clarity, such as transparent electrodes, optical coatings, and sensors. Additionally, some polymers exhibit electroluminescent properties, enabling their use in light-emitting devices and displays [42].

To summarize, polymer organic materials continue to drive innovation and advancement in materials science, offering a blend of tunable properties, flexibility, and diverse functionalities. Ongoing research efforts are expanding the understanding of their intrinsic characteristics and optimizing synthesis methods to unlock new applications and improve existing technologies. As these materials further evolve, their integration into next-generation devices promises to catalyze transformative advancements across various technological fronts.

### 2.2. Optoelectronic and Energy Applications

**Figure 3:**
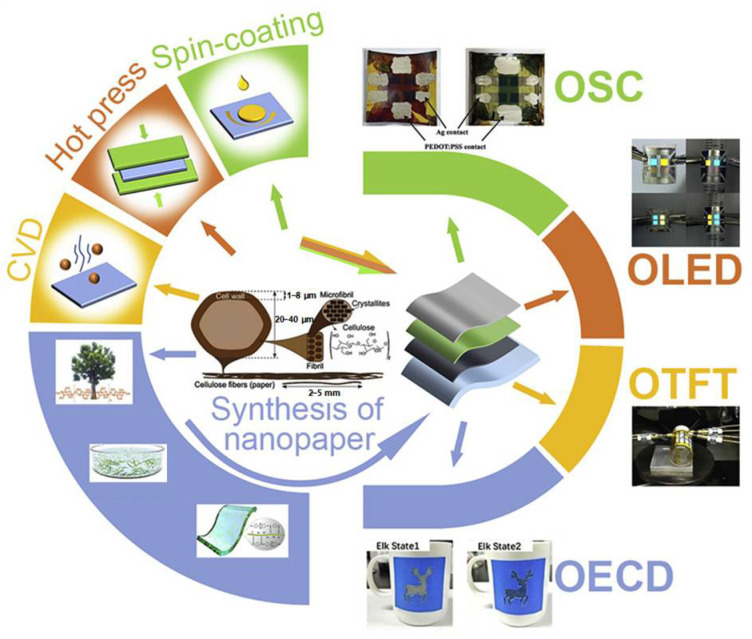
Utilization of polymer materials across diverse applications. Reprinted figure with permission from [43]. Copyright (2012) Elsevier.

As shown in Figure 3, the advancements in optoelectronic and energy applications have been significantly driven by the development and utilization of organic materials, particularly organic polymers. These materials have become central to a new generation of technologies due to their unique properties, such as flexibility, tunability, and potential for low-cost production. The remarkable progress in this field can be attributed to breakthroughs in material science, device engineering, and fabrication techniques, all of which have collectively pushed the boundaries of what is possible in optoelectronics and energy applications.

Organic photovoltaic cells (OPVs) have been at the forefront of this revolution. These devices leverage the light-absorbing capabilities of organic polymers to convert solar energy into electrical energy. The basic operation of OPVs involves several critical processes, starting with the absorption of light, which generates excitons (bound electron–hole pairs). These excitons must then diffuse to the interface between electron donor and acceptor materials, where they dissociate into free charge carriers. The free carriers are subsequently transported to the respective electrodes, producing an electric current. The efficiency of OPVs, quantified by their power conversion efficiency (PCE), depends on factors such as the absorption spectrum of the organic materials, exciton diffusion length, charge carrier mobility, and the efficiency of charge separation at the donor–acceptor interface. Recent advancements have led to OPVs with significantly improved PCE, making them viable candidates for large-scale renewable energy solutions.

Organic light-emitting diodes (OLEDs) represent another major advancement in optoelectronics, offering superior display technology and lighting solutions. OLEDs operate on the principle of electroluminescence, where light is emitted from an organic material in response to an electric current. The emission process in OLEDs involves the injection of electrons and holes from the electrodes into the organic layers, where they recombine to form excitons. These excitons then decay radiatively, emitting light. OLEDs are distinguished by their ability to produce high-quality light with excellent color accuracy and contrast. Advances in material science have led to the development of OLEDs with high efficiency and long operational lifetimes. The flexibility and thin-film nature of OLEDs enables their use in innovative applications, such as flexible displays, transparent screens, and wearable devices. The continuous improvement in OLED technology has made it a dominant player in the display market, with applications ranging from smartphones and televisions to lighting and signage.

The field of organic transistors, including organic field-effect transistors (OFETs) and organic phototransistors, has also seen substantial progress. These devices capitalize on the charge transport properties of organic polymers for applications in flexible electronics, sensors, and photodetectors. OFETs operate by modulating the conductivity of an organic semiconductor channel through an applied electric field, making them essential components in flexible electronic circuits. Recent advancements in OFETs have focused on enhancing charge carrier mobility and stability, resulting in devices that can compete with their inorganic counterparts in terms of performance. Organic phototransistors, which respond to light by generating a photocurrent, are crucial for applications in imaging, sensing, and optoelectronic integration. The ability to fabricate these devices on flexible substrates using low-cost printing techniques has opened new possibilities for large-area electronics and wearable technologies.

In addition to these device-specific advancements, there have been significant improvements in the fundamental understanding of the physical processes governing optoelectronic devices. The study of light absorption, emission, charge and energy transfer, and recombination processes in organic polymers has provided critical insights that have informed the design and optimization of devices. For example, understanding the mechanisms of exciton generation and dissociation has led to the development of materials with optimized energy levels and enhanced charge separation efficiency. Similarly, advances in controlling charge carrier mobility and minimizing recombination losses have been pivotal in achieving high-performance OPVs and OLEDs. The advancements in optoelectronic and energy applications driven by organic materials represent a significant leap forward in the field of electronics and photonics. The potential for large-scale, cost-effective production of organic devices using solution-based processes like spin-coating, inkjet printing, and roll-to-roll processing remains a significant advantage, positioning organic optoelectronics as a key player in future technology.

The prospects of organic optoelectronics and energy applications are promising. Ongoing research aims to develop new materials with superior properties, such as higher charge carrier mobility, a broader absorption spectrum, and improved stability. Additionally, the exploration of new device architectures and fabrication techniques continues to push the envelope of what is achievable. The unique properties of organic polymers, combined with continuous innovation in material science and device engineering, have enabled the development of high-performance, flexible, and cost-effective devices. As research continues to advance, the impact of organic optoelectronics on technology and society is expected to grow, offering sustainable and versatile solutions for a wide range of applications.

## 3. Material Properties and Physical Studies of Organic Polymers

Material properties and physical studies of organic polymers have become a focal point in the realm of optoelectronics due to their distinctive characteristics and potential applications. These polymers are defined by their ability to absorb and emit light, facilitating crucial processes such as charge and energy transfer. One of the fundamental properties of organic polymers is their light-absorption capability. When these materials are exposed to light, they generate excitons—bound states of electrons and holes—that play a pivotal role in the conversion of light into electrical or luminescent energy. The efficiency of exciton generation and the subsequent processes of diffusion and dissociation are profoundly influenced by the molecular structure and electronic properties of the polymer.

### 3.1. Light Absorption

The light-absorption properties of polymers are integral to their functionality in various optoelectronic applications, including organic photovoltaics (OPVs) and light-emitting diodes (LEDs). The optical absorption spectra of polymer molecules often exhibit intricate patterns due to the coupling between electronic excitations (excitons) and the vibrational modes of the molecule. These vibrational modes include, for example, C–C stretching and C–H wagging in benzene rings. The interaction between these modes influences the absorption characteristics significantly.

#### 3.1.1. Exciton–Vibrational Coupling

**Figure 4:**
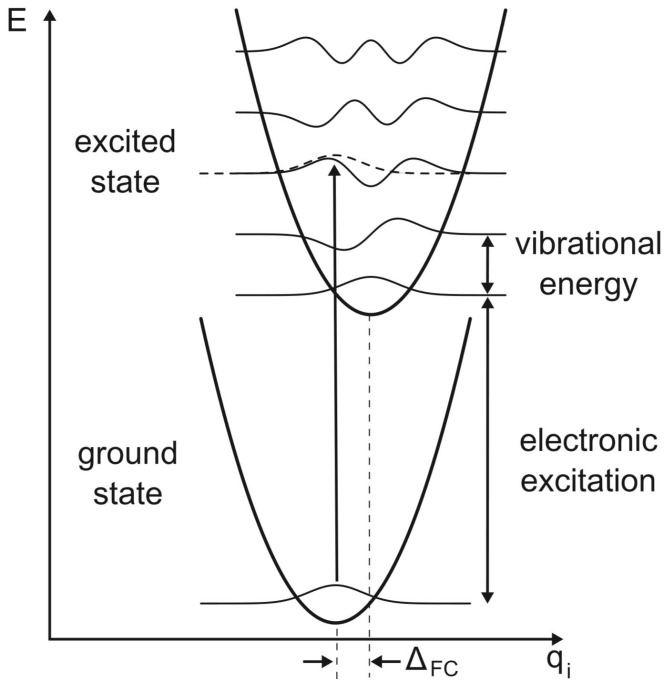
The Franck–Condon principle of light absorption in matter; electronic transitions occur between a ground state and an excited state. Reprinted figure with permission from [44]. Copyright (2013) American Physical Society.

When a polymer molecule absorbs a photon, it undergoes a transition from the ground state to an excited state. This process is not merely an electronic transition but also involves vibrational excitations. The coupling between electronic and vibrational states is quantified by the Franck–Condon principle (Figure 4), which states that the intensity of an absorption line is proportional to the overlap integral of the vibrational wavefunctions of the ground and excited states. The probability of an electronic transition that also involves vibrational excitation can be described by the Franck–Condon factor, which is the square of the overlap integral of the vibrational wavefunctions. Mathematically, this factor influences the absorption cross-section and thus the intensity of the absorption peaks observed in the spectrum.

#### 3.1.2. Vibrational Energy and Thermal Energy

The behavior of vibrational modes in the context of light absorption is largely determined by their energy relative to thermal energy. For vibrational energies significantly higher than thermal energy (*ℏω*_m_ ≫ *k*_B_*T*), the probability of transitioning from the ground state’s zeroth vibrational level to the *m*th vibrational level of the excited state can be described by a Poisson distribution. This is given by [45]

(1) $$P(m)=\frac{S^{m}e^{-S}}{m!}$$

where *S* is the Huang–Rhys factor, a dimensionless quantity representing the strength of the exciton–vibrational coupling, and *m* is the vibrational quantum number. This distribution describes how vibrational energy levels are populated during the absorption process, resulting in distinct vibrational sidebands in the absorption spectrum.

#### 3.1.3. Absorption Spectrum Features

The absorption spectrum of a polymer typically features a series of peaks corresponding to transitions involving different vibrational modes [46]. These peaks provide insights into the vibronic structure of the polymer. The position and intensity of these peaks can be used to infer the coupling strength between electronic states and specific vibrational modes, as well as the nature of the vibrational modes themselves.

The absorption process in polymers can be visualized by considering the potential energy surfaces of the ground and excited states. Upon photon absorption, the molecule is excited vertically (according to the Franck–Condon principle) to a higher electronic state, where it initially retains the vibrational configuration of the ground state. This leads to a higher probability of transitions that involve changes in the vibrational state, reflecting the nature of the potential energy surfaces involved.

#### 3.1.4. Quantitative Analysis of the Absorption Coefficient in Polymers

For a more precise understanding, the absorption coefficient *α*(*ω*) of a polymer can be described by integrating over the electronic transition dipole moment *μ* and the vibrational overlap integrals [47]:

*α*(*ω*) ∝ ∑*_i,f_* ∣⟨*ψ*_f_∣*μ*∣*ψ*_i_⟩∣^2^ *δ*(*E*_f_ − *E*_i_ − *ℏω*) (2)

where *ψ*_i_ and *ψ*_f_ are the initial and final vibronic states, respectively, and *δ* is the Dirac delta function ensuring energy conservation. This equation underscores the dependency of the absorption coefficient on both the electronic transition dipole moment and the vibrational structure.

#### 3.1.5. Practical Implications

Understanding the light-absorption characteristics of polymer materials is essential for optimizing their performance in optoelectronic devices. For instance, in organic photovoltaic cells (OPVs), the polymer’s ability to absorb light across a broad spectrum is a key determinant of solar energy conversion efficiency. By tailoring the molecular structure of polymer materials to enhance specific vibrational modes and improve exciton–vibrational coupling, it is possible to achieve more efficient light absorption, thereby increasing overall device performance [48]. For instance, in organic photovoltaic cells (OPVs), the polymer’s ability to absorb light across a broad spectrum is a key determinant of solar energy conversion efficiency [49,50].

The interplay between electronic excitations and vibrational modes governs the light-absorption properties of polymers. Leveraging principles like the Franck–Condon principle and related mathematical frameworks allow researchers to understand and optimize these properties effectively. This understanding enhances the design and functionality of polymer-based optoelectronic devices, leading to significant advancements in various applications, including solar energy harvesting and light emission.

Engineering polymers with tailored absorption characteristics can open up new possibilities in organic electronics. This capability drives advancements not only in solar energy applications but also in light-emitting devices and other optoelectronic technologies. As a result, these insights into polymer light absorption have profound practical implications, fostering the development of more efficient, high-performance devices in the field of organic electronics.

### 3.2. Light Emission

When a polymer absorbs a photon, it undergoes various relaxation processes to return to its ground state. These processes can be broadly categorized into radiative and nonradiative pathways. Understanding these mechanisms is crucial for applications in optoelectronic devices such as organic light-emitting diodes (OLEDs).

#### 3.2.1. Nonradiative Processes

Nonradiative relaxation involves the dissipation of energy through mechanisms that do not emit light, including energy dissipation, such as heat, collisions, and molecular conformational changes [51]. Energy dissipation as heat occurs when excess energy is transferred to the surrounding lattice or medium, resulting in thermal vibrations [52,53]. Collisions involve interactions with other molecules that transfer energy and facilitate relaxation. Molecular conformational changes happen when the polymer undergoes structural rearrangements, releasing energy without photon emission. These processes compete with radiative pathways and can significantly impact the efficiency of light emission in polymers.

#### 3.2.2. Radiative Processes

**Figure 5:**
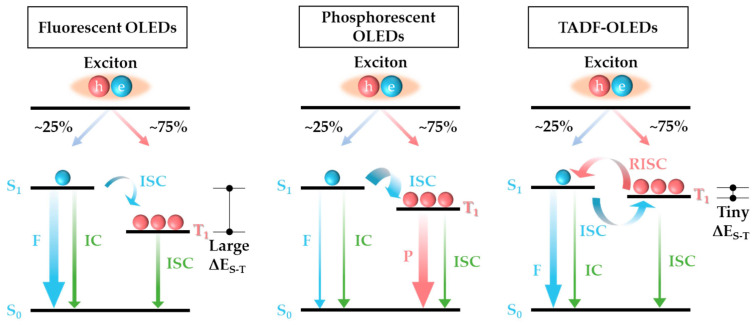
The operating principles of fluorescence, phosphorescence, and thermally activated delayed fluorescence. This figure is adapted from [55] (reproduced under terms of the CC-BY license).

Radiative relaxation involves the emission of photons, contributing to the visible light output of the material. As shown in Figure 5, the main radiative processes include fluorescence, phosphorescence, and delayed fluorescence [54].

(1) Fluorescence: Fluorescence is the emission of light from the singlet excited state S_1_ to the ground state S_0_. This process typically follows internal conversion, a rapid relaxation within the same spin multiplicity (e.g., *S*_n_ to *S*_1_). Due to the quick nature of internal conversion, fluorescence emission usually occurs from the lowest vibrational level of the excited state, following Kasha’s rule. The fluorescence emission spectrum often mirrors the absorption spectrum due to the similar vibronic transitions involved.

The intensity of fluorescence can be described by [56,57]

*I*_fluorescence_∝∣⟨*ψ*_f_∣*μ*∣*ψ*_i_⟩∣^2^ (3)

where *μ* is the transition dipole moment, and *ψ*_i_ and *ψ*_f_ are the initial and final vibronic states, respectively.

(2) Phosphorescence: Phosphorescence involves the emission from a triplet excited state *T*_1_ to the singlet ground state *S*_0_. This process is generally slower than fluorescence due to the spin-forbidden nature of the transition. Phosphorescence occurs after intersystem crossing, where the molecule transitions from *S*_1_ to *T*_1_, or through singlet fission, where *S*_0_ + *S*_1_→*T*_1_ + *T*_1_ [58].

(3) Delayed fluorescence: Delayed fluorescence is a process where the molecule first undergoes intersystem crossing to the triplet state *T*_1_, then reverts back to the singlet state *S*_1_ before emitting a photon. This can occur through thermally activated delayed fluorescence (TADF) or other mechanisms [59].

#### 3.2.3. Spectroscopic Analysis

Analyzing the emission spectra of polymers provides insights into their electronic and vibronic structures. The emission spectrum of a polymer, particularly the *S*_1_ → *S*_0_ transition, allows researchers to investigate the ground state’s vibronic details. Photoluminescence (PL) measurements are crucial for characterizing these emissions.

The photoluminescence intensity *I*_PL_ can be described by [56,60]

*I*_PL_∝∑*_i,f_* ∣⟨*ψ*_f_∣*μ*∣*ψ*_i_⟩∣^2^ *δ*(*E*_f_ − *E*_i_ − *ℏω*) (4)

where *δ* is the Dirac delta function ensuring energy conservation, and *ℏω* is the energy of the emitted photon.

#### 3.2.4. Practical Implications

Understanding light emission in polymers is essential for designing efficient OLEDs and other optoelectronic devices. In OLEDs, a high fluorescence quantum yield and appropriate spectral properties are critical for achieving bright and color-pure emission. Tailoring the molecular structure of polymers to optimize radiative processes while minimizing nonradiative losses is a key strategy for improving device performance [59,61].

To summarize, the study of light emission in polymers involves examining the complex interplay between radiative and nonradiative processes. By elucidating these mechanisms and their impact on the emission properties, researchers can design and optimize materials for advanced optoelectronic applications. This understanding leads to the development of more efficient and effective light-emitting devices, contributing to advancements in technology and industry.

### 3.3. Charge and Energy Transfer

Charge and energy transfer processes play pivotal roles in various physical and biological phenomena, driving fundamental interactions and enabling critical functionalities. Among these mechanisms, Förster Resonance Energy Transfer (FRET) stands out prominently. FRET involves the non-radiative transfer of energy from an excited donor molecule to an acceptor molecule through dipole–dipole coupling, occurring over nanometer distances. This phenomenon is heavily reliant on the spectral overlap between the donor emission and acceptor absorption spectra, as well as their relative spatial orientation.

In contrast, Dexter Energy Transfer involves a more direct exchange of energy between donor and acceptor molecules through electron-exchange mechanisms, often occurring within van der Waals distances. This process is mediated by electron wavefunction overlap and is highly dependent on the donor–acceptor spatial proximity and their respective electronic states.

**Figure 6:**
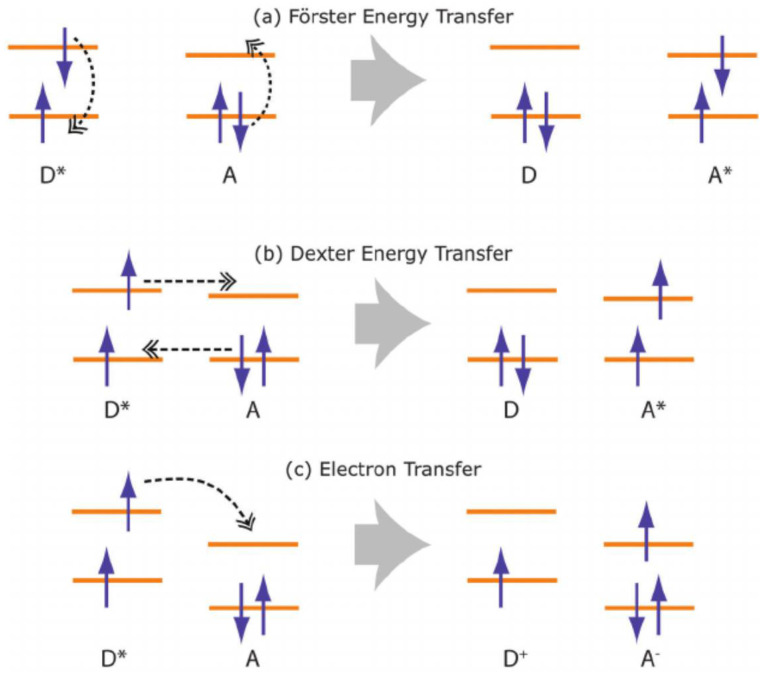
Illustration of energy- and charge-transfer mechanisms: (**a**) Förster energy transfer, (**b**) Dexter energy transfer, and (**c**) electron transfer from an excited donor molecule. Reprinted figure with permission from [62]. Copyright (2015) The Royal Society of Chemistry.

Electron transfer, another essential process, involves the movement of an electron from a donor molecule to an acceptor molecule. This transfer can occur via different mechanisms, including through a bridge molecule or directly through space. It underpins numerous biological processes, such as photosynthesis and respiration, as well as technological applications like solar cells and electronic devices. An illustration of these energy- and charge-transfer mechanisms is provided in Figure 6.

#### 3.3.1. Förster Resonance Energy Transfer (FRET)

**Figure 7:**
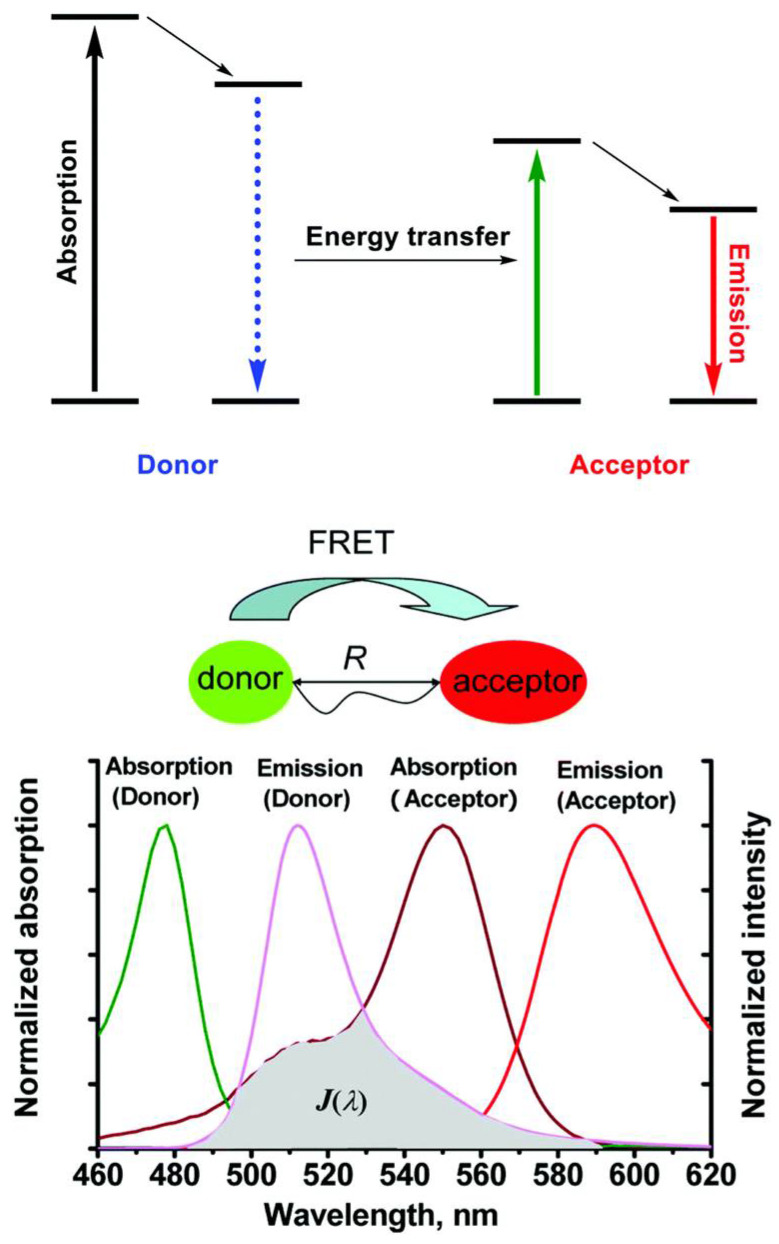
The mechanism of Förster resonance energy transfer (FRET). Reprinted figure with permission from [63]. Copyright (2013) American Chemical Society.

As shown in Figure 7, FRET operates via a dipole–dipole interaction, making it a long-range process as it involves electromagnetic fields rather than direct electron exchange. This method of energy transfer is most effective for singlet excitons, though there are instances in phosphorescent materials where FRET can facilitate triplet-state energy transfer. The efficiency of FRET depends on the overlap between the emission spectrum of the donor and the absorption spectrum of the acceptor. The FRET transfer rate *k*_FRET_ in three-dimensional materials decays with the sixth power of the distance *r* between the donor and acceptor molecules, as described by [56]

*k*_FRET_ ∝1/*r*^6^ (5)

This distance dependence underscores the necessity for close proximity between donor and acceptor molecules to ensure efficient energy transfer [64].

#### 3.3.2. Dexter Energy Transfer

In contrast to FRET, Dexter energy transfer is a short-range process that involves direct electron exchange between donor and acceptor molecules. Dexter transfer is particularly relevant for triplet excitons and requires significant orbital overlap, thus limiting its effective range to approximately 10 Å. The Dexter transfer rate *k*_Dexter_ decays exponentially with the distance *r* [57,65]:

*k*_Dexter_ ∝ e^−*r/*^*^L^* (6)

where *r* is the distance between the donor and acceptor, and *L* is a characteristic length scale associated with the decay of the electronic wavefunction overlap. Dexter transfer is thus exponentially dependent on distance, making it effective only over short ranges (typically less than 1 nm).

#### 3.3.3. Charge-Transfer Mechanisms

**Figure 8:**
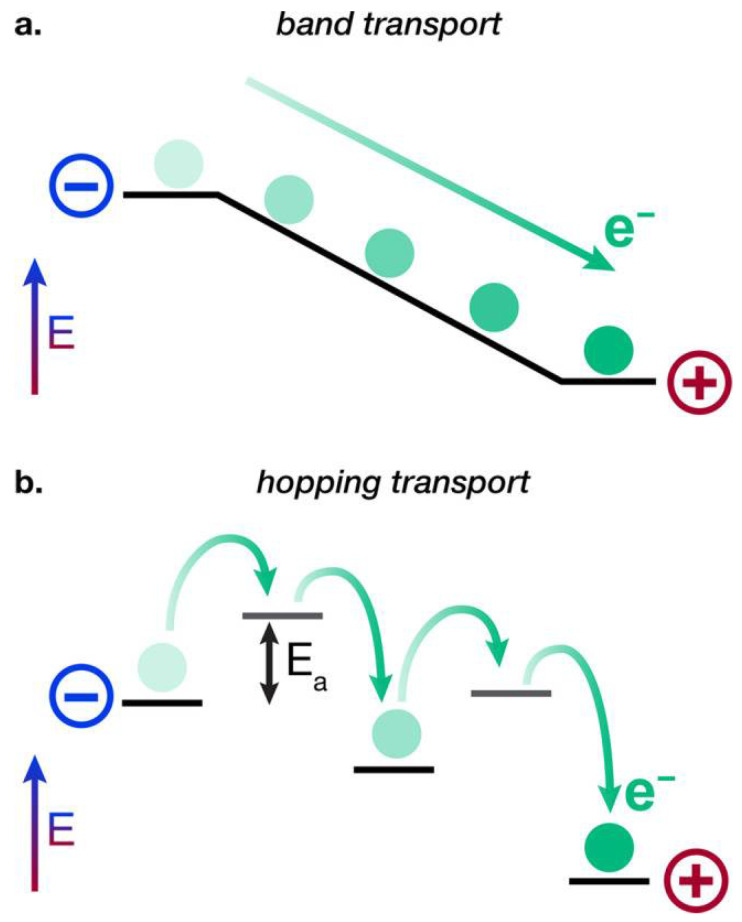
Diagrammatic depictions of (**a**) charge transport resembling ballistic bands and (**b**) charge transport via hopping mechanism. Reprinted figure with permission from [66]. Copyright (2020) American Chemical Society.

Apart from energy transfer, the transport of charges (electrons and holes) in polymers is vital for the operation of devices such as organic solar cells and light-emitting diodes. Charge transfer in polymers can occur through hopping mechanisms or band-like transport, depending on the degree of order within the material (Figure 8).

Band-like Transport: In highly ordered or crystalline regions of polymers, charge carriers can move more freely, akin to electrons in a conventional semiconductor band structure. The mobility *μ* in this regime can be significantly higher compared to hopping transport and is influenced by the presence of phonons and impurities.

Hopping Transport: In disordered polymers, charge carriers typically move via hopping between localized states. The hopping rate *k*_hop_ can be described by the Miller–Abrahams expression [67]:

(7) $$k_{\mathrm{hop}}=\nu_{0}\;\exp\;(\frac{-2r}{L})\;\exp\;(\frac{-\Delta E}{k_{B}T})$$

where *ν*_0_ is the attempt to jump frequency, *r* is the distance between hopping sites, *L* is the localization length, Δ*E* is the energy difference between initial and final states, *k*_B_ is the Boltzmann constant, and *T* is the temperature.

#### 3.3.4. Practical Implications

Understanding and optimizing energy- and charge-transfer processes are crucial for the development of high-performance polymer-based devices. For instance, in organic photovoltaics (OPVs), efficient exciton dissociation at the donor–acceptor interface is essential for generating free charge carriers that contribute to the photocurrent. Similarly, in OLEDs, efficient energy transfer from host to guest molecules within the emissive layer is vital for achieving high luminance and color purity. Advancements in material design, such as incorporating conjugated polymers with tailored energy levels and morphologies, can enhance these transfer processes. Additionally, controlling the molecular packing and domain sizes within polymer blends can significantly impact the efficiency of both Förster Resonance Energy Transfer (FRET) and Dexter transfer mechanisms, ultimately influencing the overall device performance. Therefore, practical implications of these insights include the ability to create more efficient and reliable OPVs and OLEDs, paving the way for improved renewable energy solutions and superior display technologies.

### 3.4. Charge Trapping and Recombination

**Figure 9:**
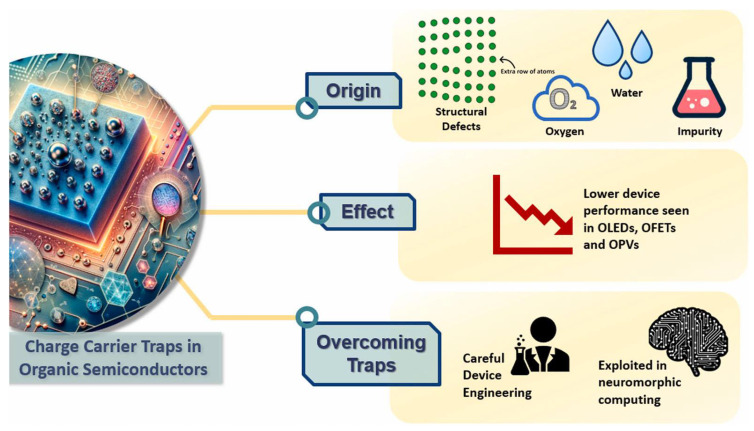
Illustration elucidating the phenomenon of charge carrier traps in organic materials. Reprinted figure with permission from [68]. Copyright (2024) Elsevier.

As illustrated in Figure 9, charge trapping significantly influences the dynamics of charge carriers in polymer-based devices, resulting in reduced mobility and the formation of space-charge regions. The nature and impact of charge traps have been extensively studied, highlighting their critical role in determining the performance of optoelectronic devices. Charge traps can originate from structural or chemical defects, contributing energy states within the band gap that can hinder carrier mobility, act as recombination centers, or lead to space-charge field formation.

#### 3.4.1. Nature of Charge Traps

Charge traps can be classified based on their origin: intrinsic (structural) or extrinsic (chemical). Intrinsic traps are associated with the inherent structural imperfections of the material, such as point defects, dislocations within crystalline domains, grain boundaries, and molecular displacements in the crystalline lattice. In polymer materials, the structural complexity often results in a more intricate landscape of intrinsic traps compared to crystalline materials.

Extrinsic traps, on the other hand, arise from chemical impurities or defects introduced during synthesis or through environmental exposure. These include intentional or unintentional dopants, synthesis byproducts, oxidation products from reactions with oxygen, and complexes formed with water or organic solvents. For example, impurities such as quinones in pentacene, oxidation products, and hydrated oxygen complexes can act as charge traps, affecting device performance.

#### 3.4.2. Effects on Charge Transport

The impact of charge traps on charge transport depends on the trap density and depth—the energy difference between the trap level and the band edge. In field-effect transistors (FETs), shallow traps (with depths on the order of several *k*_B_*T*) reduce mobility and manifest as a gradual turn-on of the transistor. Deep traps (with depths much greater than *k*_B_*T*) can shift the threshold voltage without significantly affecting mobility or subthreshold behavior. The presence of these traps can be detrimental to the overall performance of the device [69].

In organic photovoltaics (OPVs), shallow traps impede carrier transport by capturing and releasing carriers, leading to reduced effective mobility. Deep traps enhance carrier recombination by serving as sites where electrons and holes can recombine, thus reducing the efficiency of charge separation and transport. The overall effect of traps on OPV performance is a decrease in the power conversion efficiency [70].

#### 3.4.3. Mechanisms of Charge Trapping

Charge-trapping mechanisms can be described using the capture cross-section (*σ*) and the trap density (*N*_t_). The capture rate (*R*) of carriers into traps can be expressed as [71]

*R* = *N_t_σv* (8)

where *v* is the thermal velocity of the carriers. The impact of traps can also be analyzed through their influence on the carrier lifetime (*τ*). The recombination rate (*R*_rec_) can be related to the carrier concentration (*n*) and the recombination lifetime (*τ*_rec_) as [71]

(9) $$R_{\mathrm{rec}}=\frac{n}{\tau_{rec}}$$

In this context, deep traps significantly reduce *τ*_rec_, leading to higher recombination rates.

#### 3.4.4. Characterization and Mitigation

Characterizing charge traps involves various techniques, such as deep-level transient spectroscopy (DLTS), thermally stimulated current (TSC) measurements, and photo-induced current transient spectroscopy (PICTS) [71]. These methods provide insights into the energy levels, densities, and capture cross-sections of traps, enabling a better understanding of their impact on device performance.

Mitigation strategies for charge trapping focus on improving material purity, optimizing synthesis processes, and controlling the device environment. For instance, encapsulation techniques can protect the device from environmental factors that introduce traps, such as oxygen and moisture. Additionally, the design of polymers with fewer intrinsic defects and the use of additives to passivate trap states can enhance device performance.

#### 3.4.5. Practical Implications

In photorefractive (PR) polymer composites, the accumulation of sensitizer anions (e.g., C_60_^−^) under illumination and bias has been linked to the formation of hole traps, which affect the speed of PR grating formation and lead to illumination history-dependent performance. In organic solar cells, small acceptor domains in morphology can lead to the formation of traps that significantly impact charge transport and recombination. Strategies to optimize the morphology and reduce the density of traps include the use of high-purity materials, controlled synthesis, and advanced processing techniques.

To conclude, charge trapping and recombination are critical factors that determine the efficiency and stability of polymer-based optoelectronic devices. Understanding the origins and effects of charge traps allows for the development of strategies to mitigate their impact, leading to improved device performance. Ongoing research continues to uncover new insights and innovative solutions for managing charge trapping and enhancing the functionality of polymer materials.

## 4. Optoelectronic Devices and Applications

Optoelectronic devices, including organic photovoltaics (OPVs), organic light-emitting diodes (OLEDs), and organic transistors, leverage the unique properties of organic polymers for a range of applications, demonstrating significant advancements in material performance and device efficiency. OPVs convert solar energy into electrical power through efficient exciton-dissociation and charge-transport mechanisms, making them promising candidates for sustainable energy solutions [72]. OLEDs utilize the light-emission properties of organic materials to produce high-quality displays with superior color purity and energy efficiency, suitable for both consumer electronics and lighting applications [73,74]. Organic transistors, which exploit the charge transport characteristics of organic polymers, are integral in flexible electronics and advanced sensor technologies, offering the potential for low-cost, large-area manufacturing and diverse applications in modern technology [75]. The following study underscores the versatility and potential of organic polymers in transforming optoelectronic device performance and expanding their application spectrum [70].

### 4.1. Organic Photovoltaic Cells (OPVs)

Organic photovoltaic cells (OPVs) represent a promising technology for generating electricity from sunlight using organic materials. Unlike traditional silicon-based solar cells, OPVs utilize organic molecules or polymers to convert photons into electrical current [72]. This organic approach offers several advantages, including potentially lower manufacturing costs, flexibility for diverse applications, and the ability to be fabricated using solution-based processes on flexible substrates.

#### 4.1.1. Basic Principles and Structure

**Figure 10:**
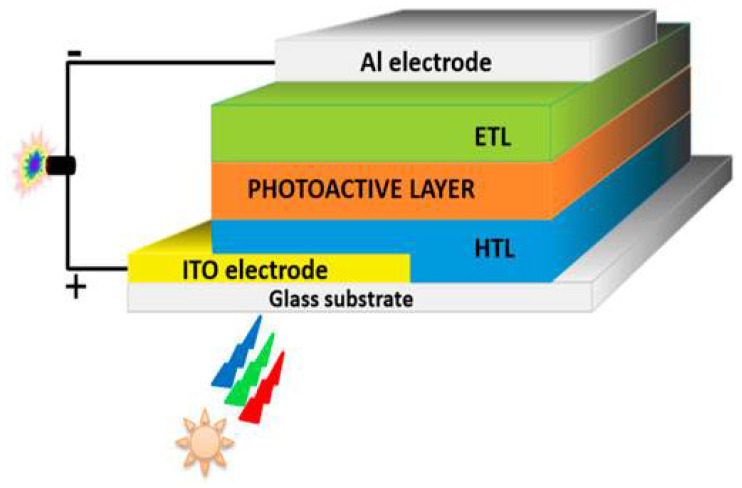
Device structure of an organic solar cell, illustrating components such as the photoactive layer for photon absorption and charge generation, charge transport layers, and electrodes. This figure is adapted from [77] (reproduced under terms of the CC-BY license).

As illustrated in Figure 10, the basic structure of an OPV typically consists of a transparent conductive substrate (such as indium tin oxide, ITO), a photoactive layer composed of a blend of electron-donating (D) and electron-accepting (A) materials, and metal electrodes (like aluminum or silver) for charge extraction. The active layer is crucial as it absorbs photons and generates excitons (bound electron–hole pairs) upon exposure to sunlight. The donor and acceptor materials are carefully chosen to facilitate efficient exciton dissociation and charge transport [76].

#### 4.1.2. Working Mechanism

**Figure 11:**
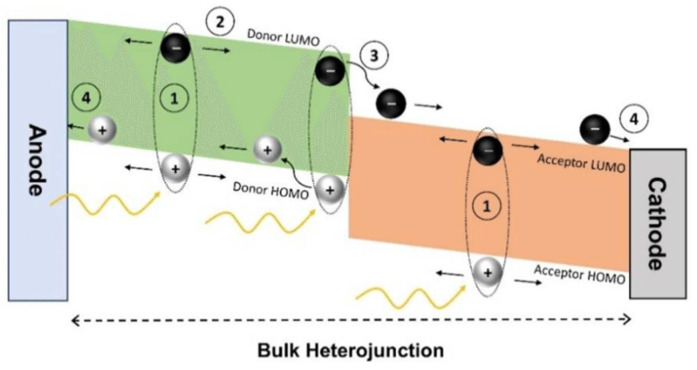
The operating mechanism of an organic solar cell. The conversion of sunlight into electric current in an organic solar cell involves four sequential stages: (1) Photon absorption, which generates excitons; (2) Diffusion of the excitons; (3) Dissociation of the excitons; and (4) Transport and collection of charges. This figure is adapted from [80] (reproduced under terms of the CC-BY license).

As demonstrated in Figure 11, the operation of OPVs begins with the absorption of photons by the photoactive layer, which generates excitons. These excitons diffuse through the photoactive layer until they reach a D-A interface, where they undergo dissociation into free electrons and holes. The electrons and holes are then transported through their respective pathways—electrons through the electron transport layer (ETL) toward the cathode and holes through the hole transport layer (HTL) toward the anode. This movement of charges generates an electric current [78]. The photocurrent density (*J*_ph_) generated by an OPV device can be described by the following equation [79]:

*J*_ph_ = *q*·*G*·*η* (10)

where *q* is the elementary charge, *G* is the incident photon flux, and *η* is the external quantum efficiency (EQE) of the device, which represents the fraction of absorbed photons that contribute to the photocurrent.

#### 4.1.3. Understanding Power Conversion Efficiency (PCE) in Organic Photovoltaic (OPV) Devices

The power conversion efficiency (PCE) of an OPV device, which quantifies its ability to convert sunlight into electricity, is given by [79,81]:

(11) $$\eta \;(\%)=\frac{J_{\mathrm{sc}}\cdot V_{\mathrm{oc}}\cdot FF}{P_{\mathrm{in}}}\times 100\%$$

where *V*_oc_ is the open-circuit voltage, *J*_sc_ is the short-circuit current density, FF is the fill factor, and Pin is the power of incident sunlight. The open-circuit voltage (*V*_oc_) of an OPV is determined by the energy difference between the highest occupied molecular orbital (HOMO) of the donor material and the lowest unoccupied molecular orbital (LUMO) of the acceptor material. Similarly, the short-circuit current density (*J*_sc_) depends on the absorption coefficient of the active layer materials and the device architecture [82]. As introduced, organic solar cells have been extensively studied due to their advantages, such as flexibility, lightweight construction, low-cost fabrication, and the potential for large-area applications. The performances of recently published organic solar cells are summarized in Table 2.

#### 4.1.4. Applications and Future Prospects

OPVs have demonstrated potential applications in various fields, including portable electronics, building-integrated photovoltaics (BIPV), and wearable devices [87]. Their lightweight and flexible nature makes them suitable for integration into curved or irregularly shaped surfaces, expanding the possibilities for solar energy harvesting. OPVs also offer the advantage of tunable optical and electronic properties through molecular engineering, enabling customization for specific applications. Future research in OPVs focuses on improving device efficiency, stability, and scalability for commercialization. Efforts include the development of new organic materials with enhanced light-absorption and charge-transport properties, optimization of device architectures to minimize energy losses, and exploration of tandem and multi-junction OPV concepts to broaden the spectral response and boost efficiency. Advances in encapsulation techniques are critical to protect OPV devices from environmental factors such as moisture and oxygen, which can degrade performance over time. Moreover, the sustainability of OPVs, given their potential for low-energy fabrication processes and use of abundant organic materials, aligns with global efforts toward renewable energy sources and reducing carbon footprints. Continued innovation and collaboration across academia, industry, and government sectors are essential to realize the full potential of OPVs as a clean and efficient technology for solar energy conversion. Therefore, OPVs represent a versatile and promising avenue for advancing solar energy technology, offering solutions that combine efficiency, flexibility, and sustainability. Continued research and development are poised to drive OPVs toward widespread commercial adoption, contributing to a more sustainable energy future.

### 4.2. Organic Light-Emitting Diodes (OLEDs)

Organic Light-Emitting Diodes (OLEDs) embody a transformative technology for producing light through organic compounds. Unlike conventional LEDs based on inorganic semiconductors, OLEDs utilize organic molecules to emit light when an electric current passes through them. This organic-based approach offers numerous benefits, such as potential cost savings in manufacturing; flexibility for various applications, including flexible displays and lighting panels; and the ability to be fabricated using solution-based processes on diverse substrates.

#### 4.2.1. Basic Principles and Structure

**Figure 12:**
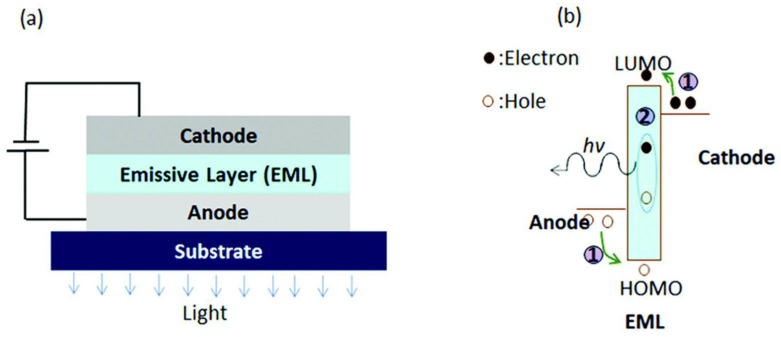
(**a**) The device configuration of single-layer OLEDs. (**b**) An illustration of the corresponding energy level diagram. The operation of an OLED can be described in two phases: (1) charge carriers (holes and electrons) are introduced from the electrode into the emissive layer, where they recombine; (2) this recombination process generates light, with its energy or color corresponding to the difference between the highest occupied molecular orbital (HOMO) and lowest unoccupied molecular orbital (LUMO) energy levels of the emissive layer. This figure is adapted from [88] (reproduced under terms of the CC-BY license).

As shown in Figure 12a, the OLED structure comprises several key components: a substrate, typically glass or plastic, providing stability; an anode made of transparent indium tin oxide (ITO) to allow light transmission; a hole-transport layer (HTL) enabling the injection of positive charge carriers (holes) from the anode into the organic layers; an emissive layer where light is generated by the recombination of electrons and holes; an electron-transport layer (ETL) facilitating the injection of negative charge carriers (electrons) from the cathode into the organic layers; and a cathode composed of a low work-function metal like aluminum, responsible for injecting electrons and completing the electrical circuit.

#### 4.2.2. Working Mechanism

OLEDs operate based on electroluminescence, where light emission occurs due to the recombination of electrons and holes within the emissive layer (Figure 12b). When a voltage is applied across the OLED structure, electrons injected from the cathode and holes injected from the anode into the organic layers move toward each other under the influence of the electric field. When an electron meets a hole within the emissive layer, they recombine, and energy released during this process is emitted as light. The color of light emitted depends on the energy bandgap of the organic material used in the emissive layer. The luminance (*L*) of an OLED device can be approximated by the following equation [88,89]:

*L* = *η*·*q*·*µ*·*V*·*F* (12)

where *η* is the quantum efficiency (the fraction of electrons that recombine to emit light), *q* is the elementary charge, *μ* is the mobility of charge carriers, *V* is the applied voltage, and *F* is the factor accounting for the ratio of holes to electrons.

#### 4.2.3. Efficiency Metrics and Device Performance

The efficiency of OLEDs is often described by the external quantum efficiency (EQE), which quantifies the number of photons emitted per injected electron–hole pair. It is given by [90,91]

(13) $$\mathrm{EQE}\;(\%)=\frac{Number\;of\;photons\;emitted}{Number\;of\;photons\;injected}\times 100\%$$

High EQE values indicate that a high percentage of injected charges are converted into emitted photons, reflecting a more efficient OLED device. OLEDs can emit light efficiently because of the organic materials’ ability to emit light directly without needing additional phosphors (unlike traditional LEDs). This direct emission results in vibrant colors and superior color reproduction, making OLEDs ideal for high-quality displays and lighting applications.

#### 4.2.4. Applications and Future Prospects

OLEDs have revolutionized display technologies and are widely used in applications such as smartphones, tablets, televisions, and wearable devices. Their advantages include high contrast ratios, wide viewing angles, fast response times, and low power consumption. OLED displays offer flexible and foldable designs due to the lightweight and bendable properties of organic materials, enabling innovative form factors and enhancing user experiences. Prospects for OLEDs involve enhancing their efficiency, lifetime, and scalability for larger-area applications. Researchers are exploring new organic materials with improved stability and performance characteristics to extend the OLED lifespan and reduce manufacturing costs. Efforts are also focused on developing efficient blue emitters, as blue OLEDs traditionally have lower efficiency and shorter lifespans compared to red and green counterparts. Furthermore, OLED technology is advancing toward new applications beyond displays, including general lighting and automotive lighting, where the thin, flexible nature of OLEDs can enable novel lighting designs and energy-efficient solutions. The potential for OLEDs to replace traditional lighting sources with more energy-efficient and environmentally friendly alternatives positions them as key players in future sustainable lighting solutions.

Overall, OLEDs represent groundbreaking technology in lighting and display industries, offering superior performance and versatility compared to conventional technologies. Continued research and development efforts are poised to further enhance OLED efficiency, durability, and applications, paving the way for broader adoption and integration into various sectors of modern technology and everyday life. OLEDs represent a transformative technology with vast potential across various sectors, from consumer electronics to lighting and beyond. Continued innovation and investment in OLED research and development are expected to drive further advancements, unlocking new applications and solidifying OLEDs as a cornerstone of future display and lighting technologies.

### 4.3. Organic Photodetectors

**Figure 13:**
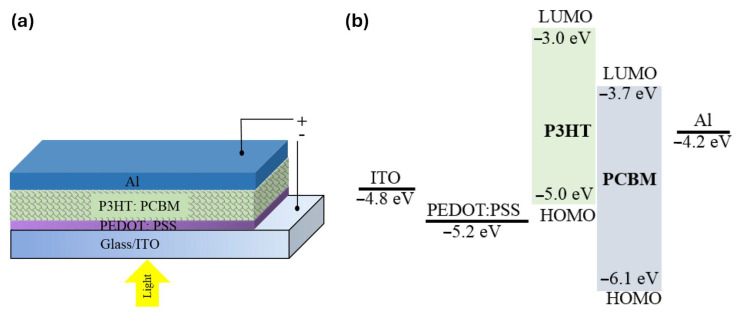
(**a**) Device configuration of the organic photodetector (OPD) and (**b**) its energy band diagram. This figure is adapted from [92] (reproduced under terms of the CC-BY license).

Organic photodetectors (OPDs) have emerged as a pivotal technology in the field of optoelectronics, leveraging organic materials to convert light into electrical signals. These devices capitalize on the unique properties of organic semiconductors, such as flexibility, lightweight construction, and the potential for low-cost, large-area fabrication. OPDs are particularly advantageous in applications where traditional inorganic photodetectors fall short, providing an array of benefits that make them suitable for a diverse range of applications from imaging to environmental monitoring. A device configuration and energy band diagram of an OPD are shown in Figure 13.

#### 4.3.1. Basic Principles and Structure

The operation of organic photodetectors is grounded in their ability to absorb photons and convert them into electrical charges. The fundamental structure of an OPD typically consists of a substrate, an active organic layer, and electrodes. The substrate can be made from materials such as glass or flexible plastics, depending on the desired application. The active organic layer, which is responsible for light absorption and charge generation, is composed of semiconducting polymers or small molecules. These materials are selected for their ability to efficiently absorb light and generate excitons, which are bound pairs of electrons and holes.

In a typical OPD structure, a transparent electrode, often made of indium tin oxide (ITO), is deposited on the substrate. This is followed by the active organic layer, which is sandwiched between the transparent electrode and a top electrode made from a metal such as aluminum or silver. The interface between the organic layer and the electrodes is crucial for the efficient extraction of generated charges, impacting the overall performance of the device.

#### 4.3.2. Working Mechanism

The working mechanism of an organic photodetector involves several key processes: light absorption, exciton generation, exciton diffusion, charge separation, and charge transport. When incident light is absorbed by the organic material, it excites the electrons from the ground state to a higher energy state, creating excitons. These excitons, due to their bound nature, need to migrate to the interface between electron-donor and electron-acceptor materials within the active layer. At this interface, the excitons dissociate into free charge carriers, namely, electrons and holes [93].

The dissociation process is driven by the energy offset between the donor and acceptor materials, which creates a built-in electric field. This field aids in the separation of the electron–hole pairs and their subsequent transport to the respective electrodes. The free carriers are then collected at the electrodes, generating a photocurrent. The efficiency of this process is influenced by several factors, including the absorption spectrum of the organic materials, the exciton diffusion length, and the charge carrier mobility.

#### 4.3.3. Understanding Responsivity and Detectivity in Organic Photodetectors

The performance of an organic photodetector can be quantified using several parameters. One of the most important is the responsivity (*R*), which measures the effectiveness of the photodetector in converting light into an electrical signal. Responsivity is defined as the ratio of the photocurrent (*I*_ph_) to the incident optical power (*P*_in_) and is given by the following equation [71]:

*R* = *I*_ph_/*P*_in_ (14)

where *I*_ph_ is the photocurrent generated by the device, and *P*_in_ is the power of the incident light. Higher responsivity indicates a more sensitive photodetector that can generate a larger photocurrent for a given amount of incident light.

Another critical parameter is detectivity (D*), which represents the ability of the photodetector to detect weak light signals. Detectivity is defined as [94,95]:

(15) $$D^{*}=\frac{R}{\sqrt{2qJ_{d}}}$$

where *q* is the elementary charge, and *J*_d_ is the dark current density (the current per unit area flowing through the device in the absence of light). High detectivity is desirable for applications requiring the detection of very low light levels, as it indicates a photodetector with low noise and high sensitivity. The performance of organic photodetectors with various active layers is summarized in Table 3.

#### 4.3.4. Applications and Future Prospects

Organic photodetectors find applications in a wide array of fields due to their unique properties. In imaging systems, OPDs are used in digital cameras and sensors to detect light and convert it into electrical signals, enabling high-resolution image capture. Their flexibility and lightweight nature make them particularly suitable for integration into wearable devices and flexible electronics, providing new possibilities for health monitoring and environmental sensing. In telecommunications, OPDs are employed in optical communication systems for high-speed data transmission and reception. Their ability to operate at various wavelengths, including the infrared spectrum, makes them ideal for use in optical fiber communication networks. Additionally, OPDs are used in environmental monitoring to detect pollutants and hazardous substances by measuring changes in light absorption or emission.

The prospects for organic photodetectors are promising, with ongoing research focused on enhancing their performance and expanding their applications. Efforts are being made to improve the quantum efficiency, responsivity, and detectivity of these devices through the development of new organic materials and device architectures. Advances in nanotechnology and material science are expected to play a crucial role in achieving these goals. Moreover, the integration of OPDs with other technologies, such as flexible displays and Internet of Things (IoT) devices, is anticipated to create new opportunities for innovation. The development of cost-effective and scalable fabrication techniques will further drive the adoption of organic photodetectors in various industries.

Ultimately, organic photodetectors represent a significant advancement in optoelectronic technology, offering unique advantages over traditional inorganic devices. Their flexible, lightweight, and cost-effective nature makes them suitable for a wide range of applications, from imaging and telecommunications to environmental monitoring and wearable electronics. With ongoing research and development, these devices are poised to play a crucial role in the future of optoelectronics and advanced sensing technologies. The ability to tune the absorption properties and enhance the performance of OPDs through material innovation and device engineering will continue to drive their evolution, cementing their place as a key component in next-generation photodetection systems.

### 4.4. Organic Phototransistors

**Figure 14:**
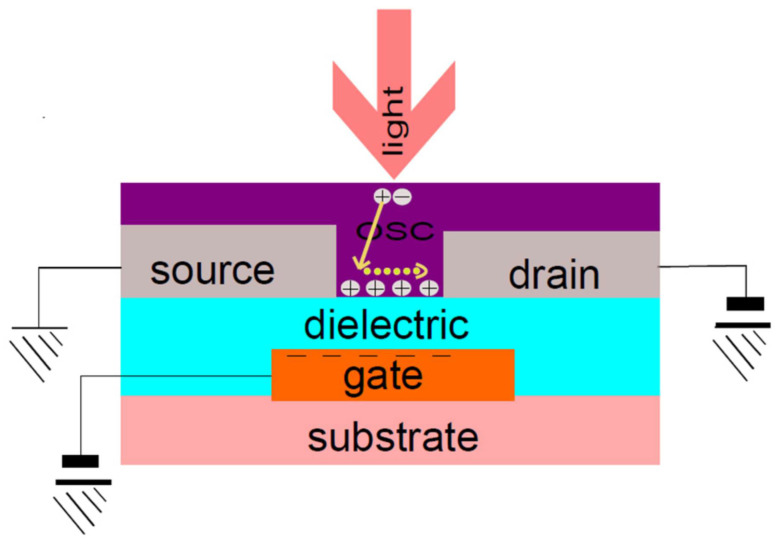
A typical organic phototransistor device structure. This figure is adapted from [99] (reproduced under terms of the CC-BY license).

Organic phototransistors (OPTs) represent a significant advancement in the field of optoelectronics, leveraging the unique properties of organic materials to detect and amplify light signals. These devices combine the functionalities of traditional transistors and photodetectors, offering enhanced sensitivity and versatility in various applications. OPTs are composed of organic semiconductors, which are carbon-based materials known for their flexibility, lightweight nature, and potential for low-cost, large-area fabrication. These characteristics make OPTs particularly attractive for a range of innovative applications, from flexible electronics to environmental monitoring and biomedical devices [99,100]. The typical device architecture of an organic phototransistor is illustrated in Figure 14.

#### 4.4.1. Basic Principles and Structure

The basic principle of an organic phototransistor involves its ability to modulate electrical current in response to light exposure. Structurally, an OPT typically consists of three layers: the gate, the dielectric, and the active organic semiconductor layer, which is placed between two electrodes known as the source and drain. The gate electrode is used to apply a voltage that controls the flow of current between the source and drain, while the dielectric layer insulates the gate from the semiconductor layer.

The active organic layer is the heart of the OPT, where light absorption and subsequent photo-induced charge generation occur. This layer is composed of organic semiconducting materials, which can be either small molecules or polymers. The choice of organic material is crucial, as it determines the OPT’s sensitivity to light, charge mobility, and overall performance. The structure of an OPT is similar to that of a field-effect transistor (FET), but with the added functionality of light sensitivity due to the organic semiconductor.

#### 4.4.2. Working Mechanism

The operation of an organic phototransistor involves several key processes: light absorption, exciton generation, charge separation, and charge transport. When light is absorbed by the organic semiconductor, it excites electrons from the valence band to the conduction band, creating electron–hole pairs known as excitons. These excitons are bound states of electrons and holes that need to be dissociated into free charge carriers to generate a photocurrent.

Upon the absorption of photons, excitons migrate to the interface between the organic semiconductor and the gate dielectric. Here, the built-in electric field, created by the applied gate voltage, facilitates the dissociation of excitons into free electrons and holes. The free electrons and holes are then transported to the source and drain electrodes, respectively, resulting in a measurable photocurrent. The gate voltage plays a crucial role in controlling the density and mobility of charge carriers within the semiconductor layer, thereby modulating the current flowing between the source and drain electrodes in response to light exposure.

#### 4.4.3. Key Performance Metrics for Organic Phototransistors

The performance of an organic phototransistor can be quantified using several parameters, such as the photocurrent (*I*_ph_), photoresponsivity (R), and external quantum efficiency (EQE). The photoresponsivity (R) measures the effectiveness of the phototransistor in converting light into an electrical signal and is defined as [101,102]

(16) $$R=\frac{I_{ph}}{P_{in}\cdot A}$$

where *P*_in_ is the incident optical power, and *A* is the active area of the phototransistor. High responsivity indicates a highly sensitive phototransistor that can generate a significant photocurrent for a given amount of incident light.

The external quantum efficiency (EQE) represents the number of charge carriers generated per incident photon and is given by [102]

(17) $$\mathrm{EQE}=\frac{R\cdot h\cdot c}{e\cdot \lambda }$$

where *h* is Planck’s constant, *c* is the speed of light, *e* is the elementary charge, and *λ* is the wavelength of the incident light. High EQE values indicate efficient conversion of photons into charge carriers, contributing to the overall performance of the OPT.

#### 4.4.4. Applications and Future Prospects

Organic phototransistors find applications in a wide range of fields due to their unique properties. In imaging systems, OPTs are used in digital cameras and sensors to detect light and convert it into electrical signals, enabling high-resolution image capture with enhanced sensitivity. Their flexibility and lightweight nature make them particularly suitable for integration into wearable devices and flexible electronics, providing new possibilities for health monitoring and environmental sensing. In telecommunications, OPTs are employed in optical communication systems for high-speed data transmission and reception. Their ability to operate at various wavelengths, including the infrared spectrum, makes them ideal for use in optical fiber communication networks. Additionally, OPTs are used in environmental monitoring to detect pollutants and hazardous substances by measuring changes in light absorption or emission.

The future prospects for organic phototransistors are promising, with ongoing research focused on enhancing their performance and expanding their applications. Efforts are being made to improve the quantum efficiency, responsivity, and detectivity of these devices through the development of new organic materials and device architectures. Advances in nanotechnology and material science are expected to play a crucial role in achieving these goals. Moreover, the integration of OPTs with other technologies, such as flexible displays and Internet of Things (IoT) devices, is anticipated to create new opportunities for innovation. The development of cost-effective and scalable fabrication techniques will further drive the adoption of organic phototransistors in various industries.

As a result, organic phototransistors represent a significant advancement in optoelectronic technology, offering unique advantages over traditional inorganic devices. Their flexible, lightweight, and cost-effective nature makes them suitable for a wide range of applications, from imaging and telecommunications to environmental monitoring and wearable electronics. With ongoing research and development, these devices are poised to play a crucial role in the future of optoelectronics and advanced sensing technologies. The ability to tune the absorption properties and enhance the performance of OPTs through material innovation and device engineering will continue to drive their evolution, cementing their place as a key component in next-generation photodetection systems.

## 5. Summary and Outlook

The exploration of organic polymers and their physical properties has unveiled a rich tapestry of possibilities for optoelectronic applications, encompassing light absorption, light emission, charge and energy transfer, and charge recombination. These materials, characterized by their flexibility, tunability, and potential for low-cost production, have demonstrated significant promise in advancing the field of optoelectronics, particularly through devices such as organic photovoltaic cells (OPVs), organic light-emitting diodes (OLEDs), and organic transistors.

Understanding the light-absorption properties of organic polymers is fundamental to their application in optoelectronic devices. The ability of these materials to absorb light and generate excitons is pivotal for the efficiency of devices like OPVs and OLEDs. The generation of excitons, which are bound electron–hole pairs, upon light absorption is the primary step in the conversion of light energy into electrical or luminescent energy. The efficiency of exciton generation and subsequent processes, such as exciton diffusion and dissociation, is critically dependent on the molecular structure and the electronic properties of the polymer. The absorption spectra of these materials can be fine-tuned through molecular design, enabling the optimization of device performance for specific applications.

The emission properties of organic polymers are equally crucial, particularly for OLEDs. Light emission in these materials occurs through radiative processes such as fluorescence and phosphorescence. Fluorescence involves the emission of light from the singlet excited state, while phosphorescence involves emission from the triplet state, typically mediated by intersystem crossing. The emission efficiency and spectral characteristics are influenced by factors such as molecular conformation, intermolecular interactions, and the presence of defects or impurities. Advances in the understanding of these processes have led to the development of highly efficient OLEDs with applications ranging from display technology to lighting.

Charge- and energy-transfer processes in organic polymers underpin the operation of a wide range of optoelectronic devices. Charge transfer involves the movement of electrons and holes within the polymer matrix, while energy transfer can occur through mechanisms such as Förster resonant energy transfer (FRET) and Dexter energy transfer. These processes are integral to the function of OPVs, where efficient charge separation and transport are required to generate an electrical current. The optimization of charge carrier mobility and the minimization of recombination losses are key to enhancing device performance. Similarly, in OLEDs, efficient charge injection and transport are essential for achieving high luminance and low power consumption.

Charge recombination is a critical process that can significantly impact the efficiency of optoelectronic devices. In OPVs, the recombination of free charge carriers can occur through various pathways, including bimolecular recombination, trap-assisted recombination, and surface recombination. Understanding the mechanisms of recombination and developing strategies to mitigate these losses is vital for improving the power conversion efficiency of OPVs. In OLEDs, charge recombination within the emissive layer is desired, as it leads to light emission. However, managing the balance of electron and hole injection and transport is crucial to ensure efficient recombination and minimize energy losses.

The practical applications of organic polymers in optoelectronic devices are vast and varied. Organic photovoltaic cells (OPVs) leverage the light-absorbing and charge-generating properties of organic polymers to convert solar energy into electrical energy. The development of OPVs has been driven by the need for sustainable and renewable energy sources, with ongoing research focused on improving their efficiency, stability, and scalability. Organic light-emitting diodes (OLEDs) utilize the light-emitting properties of organic polymers to create displays and lighting solutions with superior color quality, contrast, and energy efficiency. The flexibility and thin-film nature of OLEDs enables their use in innovative applications such as flexible displays and wearable devices. Organic transistors, including organic field-effect transistors (OFETs) and organic phototransistors, capitalize on the charge-transport properties of organic polymers for applications in flexible electronics, sensors, and photodetectors. These devices offer the potential for low-cost, large-area manufacturing, and integration into a wide range of electronic systems.

The future prospects of organic polymers in optoelectronics are promising, with ongoing advancements in material science, device engineering, and fabrication technologies. The development of new organic materials with enhanced properties, such as higher charge carrier mobility, broader absorption spectra, and improved stability, is expected to drive further improvements in device performance. Additionally, the integration of organic polymers with other advanced materials, such as perovskites and nanomaterials, holds the potential for creating hybrid devices with synergistic properties.

In summary, the study of organic polymers and their application in optoelectronic devices has opened new frontiers in the field of electronics and photonics. The unique properties of these materials, combined with their versatility and potential for low-cost production, make them ideal candidates for a wide range of applications. From renewable energy solutions like OPVs to cutting-edge display technologies with OLEDs and advanced sensing devices such as organic transistors, the impact of organic polymers on modern technology is profound. As research continues to unravel the complexities of these materials and optimize their properties, the future of optoelectronics looks brighter than ever, with organic polymers playing a central role in shaping the next generation of electronic and photonic devices.

## 6. Conclusions

The exploration and development of organic polymers have significantly advanced the field of optoelectronics, opening new avenues for various applications such as organic photovoltaic cells (OPVs), organic light-emitting diodes (OLEDs), and organic transistors. These materials, characterized by their flexibility, tunability, and potential for cost-effective production, have demonstrated exceptional capabilities in light absorption, emission, charge, and energy transfer. Understanding the fundamental processes such as exciton generation, charge separation, and recombination has been pivotal in optimizing device performance. The ability to fine-tune the absorption spectra and enhance emission efficiency through molecular design has led to the creation of highly efficient OLEDs and OPVs, showcasing the practical potential of organic polymers in creating sustainable energy solutions and innovative display technologies.

The future prospects of organic polymers in optoelectronics remain exceedingly promising, driven by ongoing advancements in material science, device engineering, and fabrication technologies. The development of new materials with enhanced properties, such as increased charge carrier mobility and broader absorption spectra, is expected to further improve the performance and stability of these devices. Furthermore, the integration of organic polymers with advanced materials like perovskites and nanomaterials offers the potential for creating hybrid devices with synergistic properties, enhancing the overall efficiency and expanding the application spectrum. As research continues to delve into the complexities of these materials and optimize their properties, organic polymers are poised to play a central role in shaping the next generation of electronic and photonic devices, making a profound impact on modern technology.

## Figures and Tables

**Table 1:** Representative material properties of various polymers are summarized.

Polymer	Physical Properties	Mechanical Properties	Optical Properties	Main Applications	Ref.
P3HT	Soluble in common organic solvents	Flexible, good film-forming	Absorbs in the visible range	Optoelectronic devices	[35]
PCBM	High electron mobility	Rigid structure	High absorption in UV-Vis range	Bulk heterojunction	[36]
PTB7	Tunable bandgap	Good tensile strength	Low optical bandgap	Optoelectronic devices	[37]
PEDOT	Conductive polymer	Flexible, processable	Transparent in the visible range	Transparent electrodes	[38]
PDCBT	High hole mobility	Strong adhesion	Good light absorption	Optoelectronic devices	[39]
CBP	High triplet energy	Rigid structure	Strong absorption in UV-Vis range	Host material for phosphorescent OLEDs	[40]

**Table 2:** Summary of recently published organic solar cell performances, including *V*_OC_, *J*_SC_, FF, and PCE.

Ref.	*V*_OC_ (V)	*J*_SC_ (mA/cm^2^)	FF (%)	Efficiency (%)
[83]	0.891	26.7	80.8	19.2
[84]	0.859	27.85	75.7	18.13
[85]	0.879	26.7	80.9	19.0
[86]	0.866	27.05	80.5	18.86

**Table 3:** A summary of organic photodetectors with various active layers.

Ref.	Active Layer	EQE [%]	R [A/W]	D [Jones]
[96]	TAPC:C_70_	56.0	0.144	2.5 × 10^13^
[97]	Co1-4Cl:PTB7-Th	68.0	0.500	1.0 × 10^12^
[98]	Poly-C60:Cy7-T	23.0	0.160	1.0 × 10^12^
[85]	PbS-QD:P3HT:PCBM	51.0	0.500	2.3 × 10^9^

## Data Availability

The data are included within the article.
